# The Impact of Carotid Artery Stenting on Cerebral Perfusion, Functional Connectivity, and Cognition in Severe Asymptomatic Carotid Stenosis Patients

**DOI:** 10.3389/fneur.2017.00403

**Published:** 2017-08-09

**Authors:** Tao Wang, Dong Sun, Yumin Liu, Bin Mei, Huagang Li, Shengming Zhang, Junjian Zhang

**Affiliations:** ^1^Department of Neurology, Zhongnan Hospital, Wuhan University, Wuhan, China

**Keywords:** carotid artery stenting, asymptomatic carotid artery stenosis, cognition, pulsed arterial spin labeling, resting-state functional MRI, default mode network

## Abstract

**Background and purpose:**

Asymptomatic carotid artery stenosis can lead to not only stroke but also cognition impairment. Although it has been proven that carotid artery stenting (CAS) can reduce the risk of future strokes, the effect of CAS on cognition is conflicting. In recent years, pulsed arterial spin labeling (pASL) MRI and resting-state functional MRI (R-fMRI) have been employed in cognitive impairment studies. For the present study, cognition is evaluated in severe asymptomatic carotid artery stenosis patients undergoing CAS, and the mechanisms underlying the cognitive change are explored by pASL MRI and R-fMRI.

**Materials and methods:**

We prospectively enrolled 24 asymptomatic, severe (≥70%), unilateral internal carotid artery stenosis patients, who were expecting the intervention of CAS. Cognition assessment (including the Montreal Cognitive Assessment Beijing Version, the Minimum Mental State Examination, the Digit Symbol Test, the Rey Auditory Verbal Learning Test, and the Verbal Memory Test) and an integrated MRI program (pASL MRI, and R-fMRI) were administered 7 days before and 3 months after CAS.

**Results:**

16 subjects completed the follow-up study. After stenting, significant improvement in the scores of the MMSE, the Verbal Memory test, and the delayed recall was found. No significant difference was found in the scores of the Montreal Cognitive Assessment Beijing Version, the Digit Symbol Test, and the immediate recall. After CAS treatment, asymptomatic carotid artery stenosis patients showed increased perfusion in the left frontal gyrus, increased amplitude of low-frequency fluctuation (ALFF) in the right precentral gyrus, and increased connectivity to the posterior cingulate cortex (PCC) in the right supra frontal gyrus. However, no significant correlations were found between these imaging changes and cognition assessments.

**Conclusion:**

Successful CAS can partly improve cognition in asymptomatic carotid artery stenosis patients. The cognition improvement may be partly attributed to the increased perfusion in the left frontal gyrus, increased ALFF in the right precentral gyrus, and increased connectivity to the PCC in the right supra frontal gyrus.

## Introduction

Carotid artery stenosis without transient ischemic attack (TIA) and stroke is considered as “asymptomatic” (1, 2). However, a number of studies have demonstrated that asymptomatic carotid artery stenosis patients had significantly poorer performance in executive function and memory, indicating that “asymptomatic” carotid stenosis might not be truly asymptomatic (3–7). Carotid endarterectomy (CEA) had been traditionally performed to prevent stroke in patients with high-grade carotid stenosis (8). However, carotid artery stenting (CAS) is now being investigated as an alternative to CEA (9). Although both CEA and CAS have been proven to reduce future strokes, the effect of CEA and CAS on cognition still remained conflicting (9–11).

In past few years, several imaging techniques, such as pulsed arterial spin labeling (pASL) MRI and resting-state functional MRI (R-fMRI), had been increasingly used to study cognitive impairment in humans. In this study, we evaluated the cognition performance in severe asymptomatic carotid artery stenosis patients undergoing CAS and explored the mechanisms underlying the cognition changes by the integrated MRI techniques including pASL MRI and R-fMRI.

## Participants, Inclusion and Exclusion Criteria

From January 2015 to June 2016, successful CAS was performed in 24 asymptomatic carotid artery stenosis patients, and the follow-up study was completed for 16 patients. No vascular complications occurred in these 16 subjects. The inclusion criteria were as follows: (1) age between 55 years and 80 years; (2) unilateral internal carotid artery stenotic degree ≥ 70%; (3) right-hand-dominant; (4) free of stroke, TIA, dementia, and depression; (5) modified Rankin Scale: 0 or 1; (6) no major medical conditions; and (7) obtained written informed consent. The exclusion criteria were as follows: (1) contralateral internal carotid artery stenosis ≥ 50%; (2) posterior circulation diseases; (3) MMSE < 26, which is a cutoff value for mild cognitive impairment; (4) functional disability (modified Rankin Scale ≥ 2); (5) severe systemic diseases and neuropsychiatric diseases (such as congestive heart failure and history of stroke); (6) any contraindications for MRI scan (e.g., metal implants); (7) low education level (<6 years); (8) evidence of carotid dissection; (9) intracranial aneurysm or arteriovenous malformation; and (10) allergy to heparin, aspirin, or clopidogrel. All procedures involved in this study were approved by Zhongnan Hospital Review Board.

## CAS Procedure and Clinical Follow-Up

After stenting, all final residual diameter stenosis was ≤20%. Systolic blood pressure was carefully maintained between 100 and 140 mmHg, and no complications were found. Aspirin and clopidogrel were continued for at least 3 months after successful intervention.

## Neurocognitive Assessment

Cognition assessments were completed within 1 week after MRI scan. The MMSE and MoCA Beijing Version were used to assess the global cognition. In the Digit Symbol Test, subjects were required to translate numbers to symbols in a given time and correct translations within 90 s were recorded. The Rey Auditory Verbal Learning Test was used to evaluate the memory and verbal learning ability. In this test, participants had to recall all the words remembered, and this procedure was repeated five times. These were recorded so that the total number of words was immediately recalled during the first five repeats and the delayed recall of the first list. In the Digit Span Test, participants were required to repeat the orally presented lists of numbers, beginning with a two-number sequence, and each correct performance was followed by one additional number. In the forward span, participants were asked to retell the span in forward order, and in the backward span, the participant was asked to retell the span in reverse order.

## Brain Imaging Acquisition

MRI images were acquired using a 3.0 T Siemens scanner in Zhongnan Hospital. T1 images were collected using MP-RAGE sequence. Scan parameters were as follows: flip angle = 9°, TR = 2,250 ms, TE = 2.26 ms, slice thickness = 1.0 mm, inversion time = 900 ms, number of slices = 176, data matrix = 215 × 256, and FOV = 256 mm × 224 mm. pASL perfusion images were collected using Q2TIPS II technique. Scan parameters were as follows: TR = 2,500 ms, TE = 11 ms, FOV = 240 mm × 240 mm, matrix = 64 × 64, FA = 90°, and slice thickness = 6 mm. R-fMRI was acquired using EPI sequence: repetition time = 2,000 ms, echo time = 30 ms, flip angle = 90°, number of slices = 33, slice thickness = 3.8 mm, gap = 1 mm, data matrix = 64 × 64, and field of view = 240 mm × 240 mm.

## Image Processing

### Pulsed arterial spin labeling

relCBF maps were automatically generated by Siemens workstation, and the relCBF correct map of each participant was normalized to EPI template provided by Statistical Parametric Mapping 8 (SPM8). The final voxel size was 3 mm× 3 mm× 3 mm. Each subject’s relCBF map was transformed into standard MNI space using these transformation parameters. The normalized relCBF maps were smoothed for comparisons with 8-mm FWHM isotropic Gaussian kernel. These individual maps were then entered into SPM8 to identify significant different regions between two groups.

### R-fMRI Preprocessing

Resting-state functional MRI preprocessing was performed with Data Processing Assistant for Resting-State fMRI (DPABI 2.1). The first 10 volumes of each time series were abandoned. Then, the images were corrected for slice timing and realigned. Afterward, images were normalized into standard MNI space and smoothed with 8-mm FWHM isotropic Gaussian kernel.

### Amplitude of Low-Frequency Fluctuation (ALFF)

Amplitude of low-frequency fluctuation calculation was performed with Resting-State fMRI Data Analysis Toolkit (REST 1.8). One-sample *t*-test was performed using SPM8 in each group to detect the regions with higher-than-mean ALFF. Two-sample *t*-test was performed to determine differences between these mALFF images. Significantly different regions were shown on MNI templates. The two-sample *t*-test results were restricted within the mask made from the results of one-sample *t*-tests performed for two groups.

### Functional Connectivity

All images were filtered with a 0.01–0.08 Hz band-pass filter to reduce the noise before FC analysis. The ROI was located in the bilateral posterior cingulate cortex (PCC). The mean ROI signal was counted by average all voxels in bilateral PCC. The ROI time course was used for correlation analysis with all other voxels in the brain. To normalize the correlation coefficients, Fisher *z*-transform was then applied. One-sample *t*-test was performed using SPM8 in each group to detect the regions with significant connectivity to the PCC. Then, two-sample *t*-test was performed to determine differences between these *z*-FC image groups. Significant different regions were shown on MNI templates. The two-sample *t*-test results were restricted within the mask made from the results of one-sample *t*-tests performed for two groups.

### Statistical Analysis

We used IBM SPSS 20.0 and SPM8 to perform statistical analyses. Paired Student’s *t*-test was used to detect significant differences, and significance was set at 0.05. Age and education were used as covariates in all tests involving cognition. After analyzing pASL, ALFF, and FC, regions with significant differences were extracted as ROIs, and Spearman correlation was then performed to detect the relationship between imaging differences and cognition scores.

## Results

Part 1: Table 1 shows the demographic information and cognitive test scores at baseline and 3 months after CAS. Significant improvements in the MMSE, Verbal Memory test, and delayed recall were observed. There is no decline in any individual scores (Table 1).

**Table 1:** Demographic information and neuropsychological test scores at baseline and 3 months after CAS.

Characteristics	Baseline	3 months after stenting	*P*-value
Age (years)	66.8 ± 5.8		
Male:female	12:4		
Education (years)	9.9 ± 3.0		
Hypertension	15		
Diabetes mellitus	4		
Hypercholesterolemia	7		
Stenotic side			
Left	5		
Right	11		
MMSE	27.0	28.0	0.01*
MoCA	23.5	24.0	0.05
Verbal Memory Test			
Forward digit span	6.0	6.0	0.04*
Backward digit span	4.0	5.0	0.04*
Rey Auditory Verbal Learning Test			
Immediate recall	29.5	34.5	0.19
Delayed recall	4.0	6.5	0.03*
Digit Symbol Test	26.5	30.5	0.14

*Statistically significant difference.

*CAS, carotid artery stenting*.

**Figure 1:**
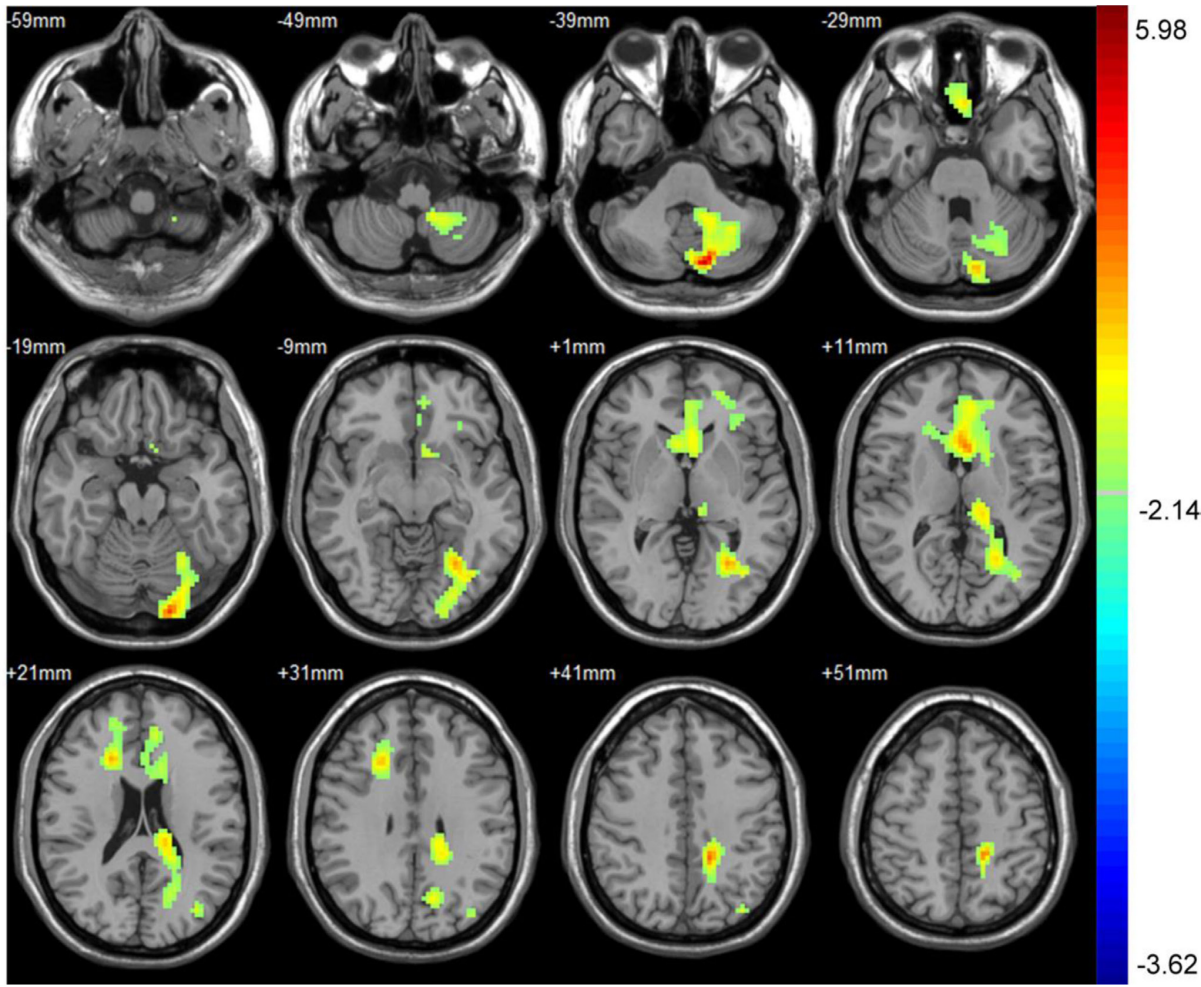
The CBF was significantly increased in the left frontal gyrus, anterior cingulate, left occipital gyrus, and left cerebellum 3 months after carotid artery stenting. The result was corrected using the AlphaSim program, which was set at *P* < 0.01 and number of voxels > 611, which was corresponded to a corrected *P* < 0.05. The left part of the figure represented the patient’s right side.

Part 2: Difference in CBF between baseline and 3 months after performance. Compared with baseline, the asymptomatic carotid artery stenosis patients showed the increased CBF mainly in the left frontal gyrus, anterior cingulate, left occipital gyrus, and left cerebellum (Table 2; Figure 1).

**Table 2:** Significant CBF difference between baseline and 3 months after CAS with their location.

	Number of voxels	Peak MNI coordinate			Peak MNI coordinate region	Peak *T* value
		*X*	*Y*	*Z*		
1	1956	−12	−81	−36	Left occipital gyrus, left cerebellum	5.98
2	1212	−3	15	12	Left frontal gyrus, anterior cingulate	3.97

*CAS, carotid artery stenting*.

**Figure 2:**
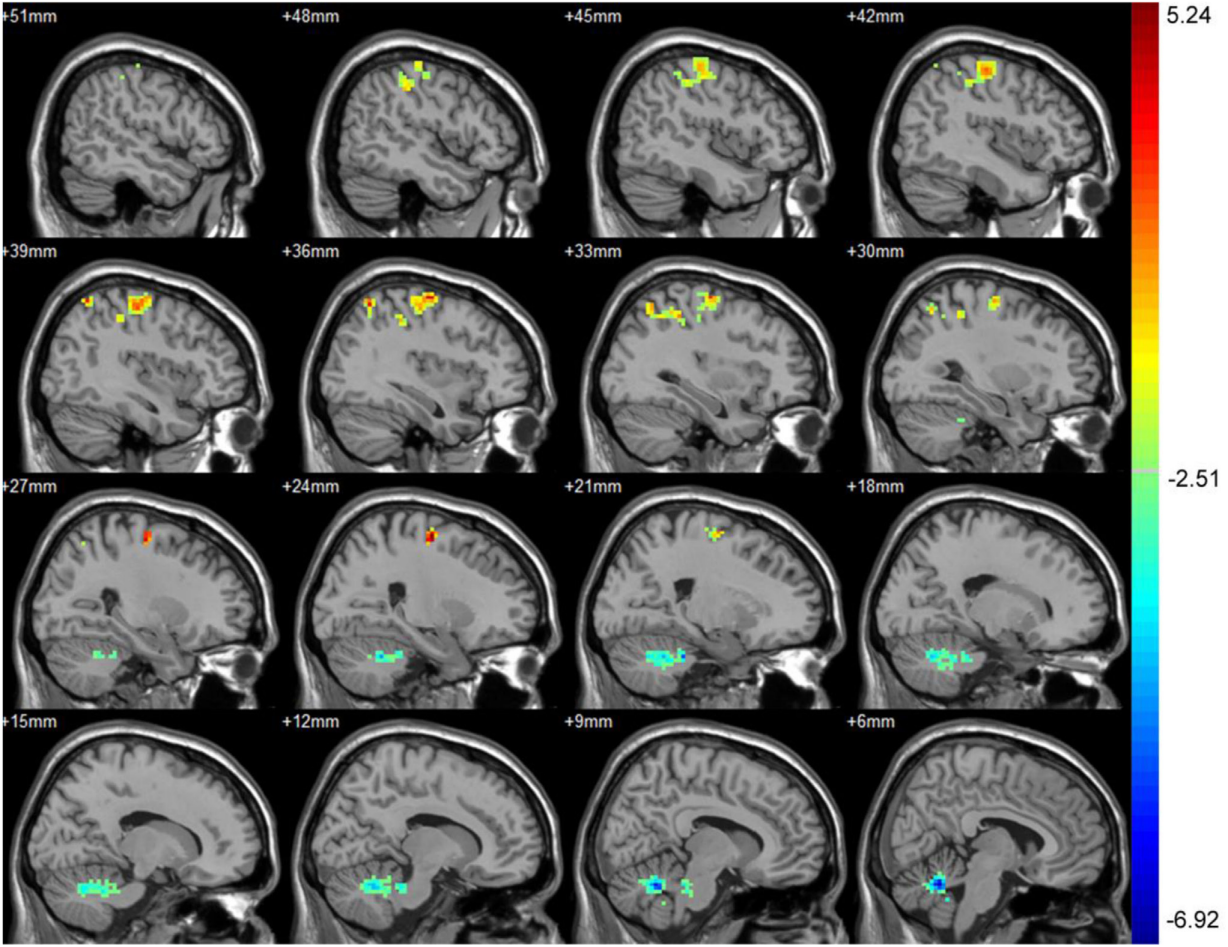
Significant difference in amplitude of low-frequency fluctuation (ALFF) between baseline and 3 months after treatment. After treatment, the asymptomatic carotid artery stenosis patients showed significantly increased ALFF predominantly in the right precentral gyrus. The asymptomatic carotid artery stenosis patients also showed decreased ALFF mainly in the left and right cerebellum anterior lobe. Red indicates 3 months after treatment > baseline and blue indicates 3 months after treatment < baseline. The result was corrected using the AlphaSim program, which was set at *P* < 0.01 and number of voxels > 175, which was corresponded to a corrected *P* < 0.025.

Part 3: Differences in ALFF between baseline and 3 months after treatment. After treatment, the asymptomatic carotid artery stenosis patients showed significantly increased ALFF predominantly in the right precentral gyrus. The asymptomatic carotid artery stenosis patients also showed decreased ALFF mainly in the left and right cerebellum anterior lobe (Table 3; Figure 2).

**Table 3:** Significant ALFF difference between baseline and 3 months after treatment with their location.

	Number of voxels	Peak MNI coordinate			Peak MNI coordinate region	Peak *T* value
		*X*	*Y*	*Z*		
1	267	24	−12	60	Right precentral gyrus	5.24
2	550	6	−57	−27	Left and right cerebellum anterior lobe	−6.92

*T > 0 indicated increased ALFF 3 months after treatment. T < 0 indicated decreased ALFF 3 months after treatment*.

*ALFF, amplitude of low-frequency fluctuation*.

**Figure 3:**
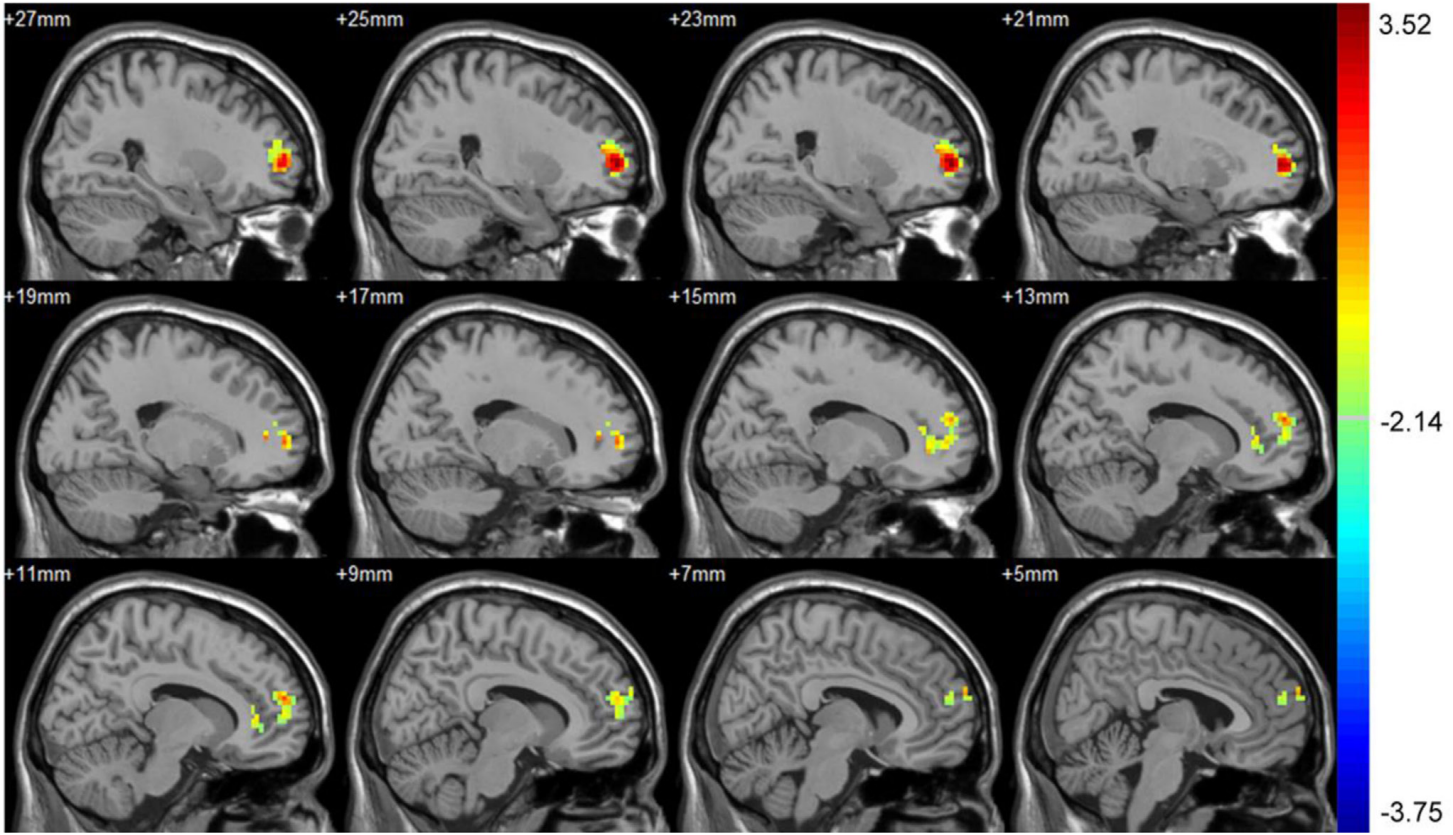
Significant connectivity difference to the posterior cingulate cortex (PCC) between baseline and 3 months after intervention. After treatment, the asymptomatic carotid artery stenosis patients showed the increased connectivity to the PCC mainly in the right supra frontal gyrus. The result was corrected using the AlphaSim program, by setting at *P* < 0.01 and number of voxels > 142, which corresponded to a corrected *P* < 0.05.

Part 4: Differences in FC to PCC between baseline and 3 months after intervention. After intervention, the asymptomatic carotid artery stenosis patients showed the increased connectivity to the PCC mainly in the right supra frontal gyrus. There was no region showing decreased connectivity to the PCC (Table 4; Figure 3).

**Table 4:** Significant connectivity difference to the posterior cingulate cortex between baseline and 3 months after intervention with their location.

	Number of voxels	Peak MNI coordinate			Peak MNI coordinate region	Peak *T* value
		*X*	*Y*	*Z*		
1	186	24	57	3	Right supra frontal gyrus	3.49

Part 5: Relationship between imaging changes and cognition scores. No significant correlation was found between imaging differences and cognition scores (*P* > 0.05 for all).

## Discussion

In this study, we found that CAS could improve global cognition (assessed by MMSE) and memory (assessed by Verbal Memory test and delayed recall test) in asymptomatic carotid artery stenosis patients, which is agreed with previous reports (12, 13). Since cognitive impairment could also predict mortality, it may be misleading that using stroke as the only outcome marker in CEA and CAS (14).

Silent infarction and cerebral hypoperfusion are the main mechanisms in the development of cognitive decline in asymptomatic carotid artery stenosis patients, indicating that cognition may be improved by reducing silent infarction and restoring CBF. Our results showed the increased regional CBF mainly in the left frontal gyrus, anterior cingulate, left occipital gyrus, and left cerebellum after CAS. The increased CBF found in the occipital gyrus and cerebellum could be explained by the presence of functional circle of Willis. Since frontal gyrus and anterior cingulate consisted of regions that mediated memory and executive function, increased CBF in these regions may contribute to the cognitive improvement after CAS.

In recent years, more and more studies had demonstrated that cognition output was not dependent on the individual brain region, but dependent on network regions (15). A number of studies had already demonstrated this opinion in patients with neurodegenerative diseases (16, 17). The default mode network (DMN) was one of them (18). By functional MRI and PET studies, the most common DMN components are the PCC, the medial prefrontal cortex, the anterior cingulate cortex, the inferior parietal lobule, and other regions (19, 20). The DMN was suggested to play important role in cognition, such as reviewing past knowledge and processing memory (18, 21–23). Another reason selecting DMN for analysis was that previous study had demonstrated that DMN was especially easily affected by hypoperfusion (24). The PCC, supplied by the precuneal artery from its origins at the ICA, had been shown to be a key point in DMN and involved in cognitive decline (19).

Among the indexes utilized in R-fMRI, ALFF was a useful index to reflect spontaneous neuronal activities (25–28). We noted that the asymptomatic carotid artery stenosis patients showed increased ALFF mainly in the right precentral gyrus after CAS, which belonged to the DMN. Thus, cognitive improvement after CAS could also be partially attributed to the increased activities in these regions. Since previous studies had indicated that the cerebellum not only regulated motor control but also involved in cognition tasks (29, 30). The decreased ALFF in the cerebellum anterior lobe may further illustrate the cognition role of cerebellum, but the mechanisms had not been illustrated exactly and needed further more studies.

We compared the FC to the PCC before and after CAS treatment and found that the asymptomatic carotid artery stenosis patients showed increased connectivity to the PCC mainly in the right supra frontal gyrus after CAS. There was no region showing decreased connectivity to the PCC. The increased regions were also overlapped with the anterior part of the DMN. Since the anterior part of the DMN was specially associated with executive function and memory, the cognitive improvement could also be partially attributed to the increased FC to the PCC in the anterior part of DMN, but no significant correlation was found (31, 32).

Nevertheless, several limitations in this study should be discussed. First, the sample size was not big enough. Second, it was extremely difficult to define the stenosis timing. Longer occlusion duration may potentially affect the cognition reversibility. Third, the follow-up interval was relatively short. Fourth, no control group was included.

In summary, this prospective study demonstrated that successful CAS could partly improve cognition dysfunction in asymptomatic carotid artery stenosis patients. By the integrated MRI techniques including pASL MRI and R-fMRI, we also found increased perfusion in the left frontal gyrus and anterior cingulate, increased ALFF in the right precentral gyrus, and enhanced connectivity to the PCC in the right supra frontal gyrus after CAS. Since these regions are overlapped with DMN, the cognition improvement by CAS in asymptomatic carotid artery stenosis patients may be partly attributed to these changes.

## Ethics Statement

This study was carried out in accordance with the recommendations of “Zhongnan Hospital Review Board” with written informed consent from all subjects. All subjects gave written informed consent in accordance with the Declaration of Helsinki. The protocol was approved by the “Zhongnan Hospital Review Board.”

## Author Contributions

TW: analysis and interpretation of data. DS, YL, BM, HL, and SZ: acquisition of data. JZ: study concept and design.

## Conflict of Interest Statement

The authors declare that the research was conducted in the absence of any commercial or financial relationships that could be construed as a potential conflict of interest.
